# M2 macrophage associated genes shape prognosis and tumor progression in human colorectal cancer

**DOI:** 10.1016/j.isci.2026.116230

**Published:** 2026-06-05

**Authors:** Meng Li, Lingling Dong

**Affiliations:** 1 Center for Genetic Medicine, the Fourth Affiliated Hospital of School of Medicine, and International School of Medicine, International Institutes of Medicine, Zhejiang University, Yiwu, Zhejiang, China; 2 College of Life Sciences, Anhui Normal University, Wuhu, Anhui, China; 3 State Key Laboratory of Reproductive Medicine and Offspring Health, Nanjing Medical University, Nanjing, Jiangsu, China

**Keywords:** Health sciences, Medicine, Oncology

## Abstract

Tumor-associated macrophages (TAMs) are key components of the colorectal cancer (CRC) microenvironment, yet the transcriptional programs associated with M2 states and their prognostic relevance remain incompletely defined. Here, single-cell RNA-seq analysis of CRC identified macrophages that were further classified into M0-like, M1-like, and M2-like states. Pseudotime analysis identified 311 genes associated with M2-like polarization. Cox and LASSO analyses yielded 16 M2-associated prognostic genes (M2Gs), whose robustness was further validated in an independent dataset. PLTP, NPL, and DCTPP1 were enriched in M2 macrophages in CRC tissues. Knockdown of these genes reduced IL-10-induced M2-like polarization, and conditioned media from knockdown macrophages suppressed the malignant phenotypes of HCT116 cells. Finally, a 16-M2G-based risk model stratified prognosis in TCGA and two independent cohorts. Collectively, our study defines an M2-like TAM-associated transcriptional signature with prognostic relevance in CRC and provides functional evidence that selected M2G modulate macrophage polarization and tumor cell phenotypes.

## Highlights

• Single-cell profiling defines M0-like, M1-like, and M2-like TAM programs in CRC
• A 16-gene M2-associated signature provides moderate, stage-independent prognosis
• Three M2Gs regulate M2-like polarization and impact CRC tumorigenicity

## Graphical abstract

**Figure:**
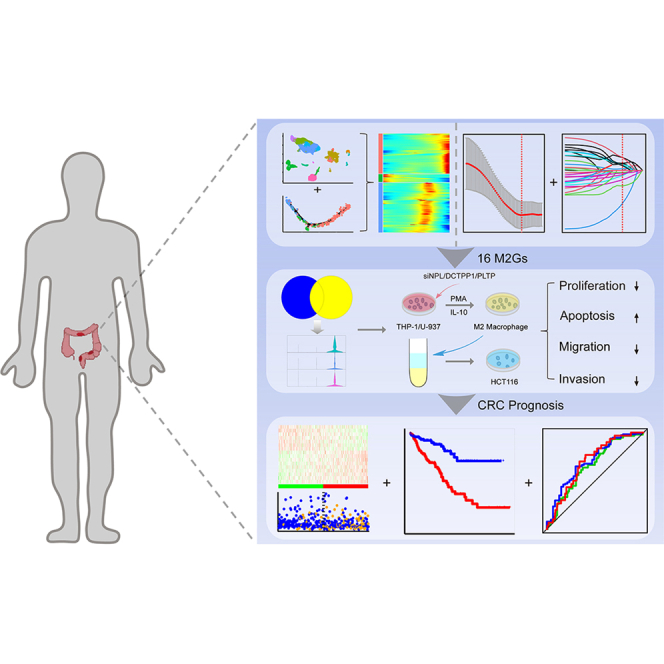

## Introduction

Colorectal cancer (CRC) accounts for about 10% of all cancer incidences and is the second leading cause of cancer-related death.^1^ As CRC is asymptomatic in early stages, it is usually diagnosed at relatively late stages and thus raises the risks to the survival of patients. Currently, the tumor-node-metastasis (TNM) stages remain an important prognostic factor in the decision of therapy strategies against CRC, while other prognostic factors have been proposed to improve the prediction accuracies.^2^ KRAS and BRAF are two important prognostic factors in CRC. KRAS was known to be a pan-cancer oncogene and its mutation was proven to be associated with poor prognosis in patients with CRC^3^^,^^4^; BRAF is a downstream target of KRAS, whose upregulation predicted poor prognosis in patients with stage II and III CRC.^5^ Importantly, accumulating evidence indicates that oncogenic KRAS/BRAF signaling does not only drive cancer cell-intrinsic proliferation and survival but also reshapes the tumor microenvironment (TME) by modulating the secretion of cytokines, chemokines, and growth factors, as well as the expression of immune regulatory molecules.^6^^,^^7^^,^^8^ These changes promote the recruitment and functional reprogramming of myeloid cells, particularly tumor-associated macrophages (TAMs), and favor their skewing toward an immunosuppressive, M2-like phenotype. Thus, genetic alterations and the immune microenvironment are functionally intertwined. Apart from these, the TME plays a key role in tumor progression, via regulating drug resistance, immune escape, cancer metastasis and so forth.^9^^,^^10^ It consists of a series of local cells, such as fibroblasts, cancer stem cells, TAMs, T cells, NK cells, extracellular matrix and so forth. It is generally believed that TAMs and their biomarkers are closely associated with tumorigenesis, metastasis, and prognosis.^11^^,^^12^

Macrophages mediate immune responses against pathogens and regulate homeostasis. In cancer, TAMs display remarkable plasticity and exist along a continuum of activation states. For conceptual clarity, they are often described in terms of two prototypical poles, classically activated (M1) and alternatively activated (M2), which underlie their apparently opposing functions in tumors.^13^ With the induction of interferon and lipopolysaccharide (LPS), M1 macrophages are activated and produce TNF-α, IL-12, and other factors executing anti-tumor function.^14^^,^^15^ Alternatively, under TGF-α and IL-4, M2 macrophages are activated and promote tumorigenesis.^16^^,^^17^ In human malignancies, most TAMs exhibit mixed or intermediate features along this continuum but are frequently biased toward an M2-like, pro-tumor program.^13^ Consistent with this concept, increasing evidence has highlighted the prognostic relevance of M2-associated transcriptional program. For example, a recent study has documented the role of M2-related genes in the prognosis of non-small cell lung cancer, which, however, remained unclear in CRC.^18^

Despite advances in immunogenomic profiling, the molecular determinants of TAM polarization and their prognostic implications in CRC remain insufficiently understood. In this study, we integrated scRNA-seq analysis with functional validation to uncover the role of macrophage polarization in CRC progression. We identified and characterized three macrophage subtypes and revealed a panel of genes associated with M2 polarization. Through Cox regression and LASSO analyses, we established 16 M2Gs that stratified patients according to survival outcomes. Importantly, we validated three representative genes, PLTP, NPL, and DCTPP1, by siRNA-mediated knockdown in THP-1 derived macrophages, demonstrating that reduced M2 polarization in turn suppressed the tumorigenic potential of HCT116 CRC cells. These findings not only highlight the importance of M2Gs in CRC prognosis but also provide mechanistic evidence linking macrophage polarization to tumor progression.

## Results

### Single-cell transcriptomic analysis of CRC reveals macrophage heterogeneity

**Figure 1:**
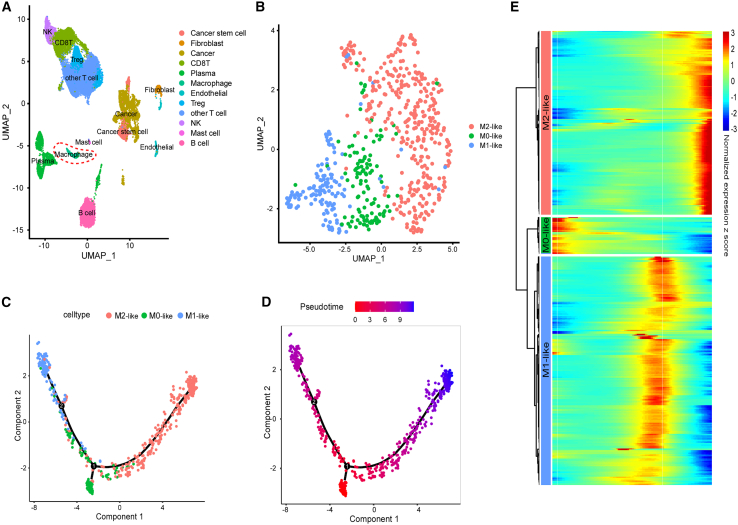
Exploration of M2 polarization-related genes in CRC scRNA-seq data (A) The UMAP plot of the whole CRC scRNA-seq data, with the macrophage subgroup, is shown in (B). (C) The trajectory of macrophage polarization was plotted according to cell types and (D) pseudotime values, respectively. (E) The heatmap showed the expression patterns that were closely correlated with the cell trajectory.

We analyzed a CRC scRNA-seq dataset comprising 28,257 cells and 23,032 genes that passed quality control. PCA and UMAP dimensionality reduction identified 23 clusters, which were annotated into 12 subgroups using established cell markers: cancer stem cells, fibroblasts, cancer cells, T cells (CD8^+^ T, Tregs, and other T cells), plasma cells, macrophages, endothelial cells, NK cells, mast cells, and B cells (Figures 1A and S1A).

To further characterize macrophage heterogeneity, we extracted this subgroup and identified seven transcriptionally distinct clusters (Figure S1B). Based on the expression of canonical activation markers, these clusters were operationally annotated as M0-like (117 cells), M1-like (135 cells), and M2-like (359 cells). In the UMAP embedding, cells annotated as M0-like were positioned between the M1-like and M2-like clusters, consistent with an intermediate activation state along a continuum from pro-inflammatory (M1-like) to immunoregulatory (M2-like) programs rather than three rigid, discrete populations (Figures 1B, S1C, and S1D). We then analyzed the genes specifically enriched in each macrophage state. S100A8, *FCN1*, S100A9, and *HSPA1B* were enriched in M0-like macrophages; *IL1B*, *PLAUR*, *CXCL8*, and *BCL2A1* were enriched in M1-like macrophages; *C1QA*, *C1QB*, *C1QC*, and *APOE* were enriched in M2-like cells (Figure S1E).

To explore TAM polarization dynamics in CRC, we performed trajectory analysis using Monocle2. As expected, M1-like and M2-like cells occupied distinct branches diverging from M0-like macrophages (Figure 1C). Pseudotime visualization showed a linear distribution of the three macrophage states (Figure 1D). Genes associated with M2 polarization were clustered in a heatmap, yielding 311 M2 polarization-related genes (Figure 1E and Table S6).

### Identification of conserved M2-associated prognostic genes

**Figure 2:**
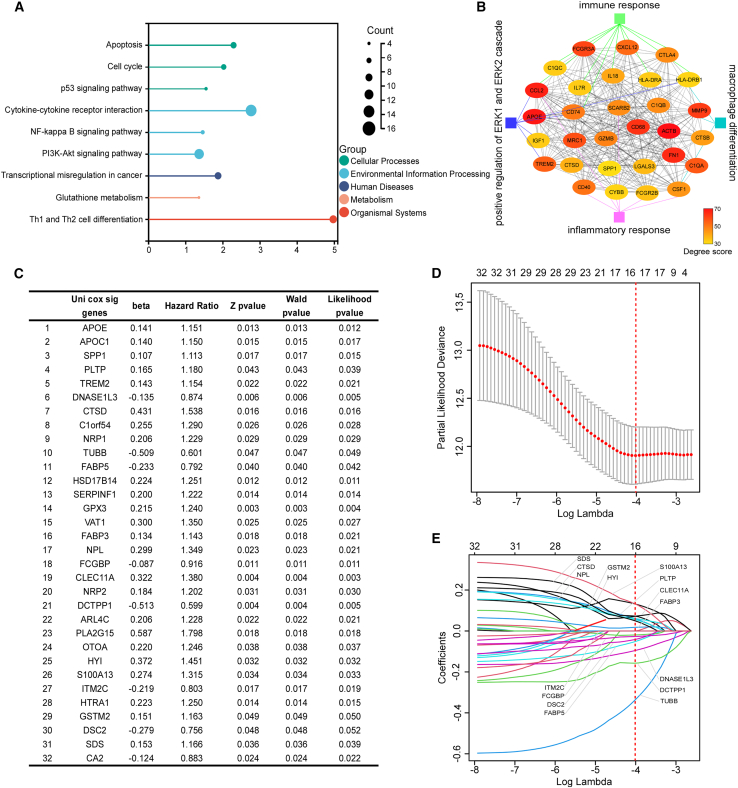
Identify 16 M2 polarization-related genes related to the colorectal cancer prognosis (A) GO analyses on the 311 M2 polarization-related genes. (B) The protein-protein interaction analysis of the top 30 proteins selected from 311 M2 polarization-related genes according to the degree scores. (C) The univariate Cox regression analysis identified 32 candidate genes that were significantly correlated with the prognosis of CRC. (D and E) The LASSO analysis was performed on the 32 candidate genes and finally resulted in 16 M2Gs that were significantly correlated with the prognosis of CRC.

To investigate the enrichment processes and pathways of these 311 M2 polarization-related genes, we performed KEGG analysis, which revealed that these genes were enriched in the Th1 and Th2 cell differentiation, Transcriptional misregulation in cancer, cell cycle, PI3K-Akt signaling pathway and so forth. (Figure 2A) Protein-protein interaction analysis was also performed to determine the interactions top 30 M2 polarization-related genes according to their degree scores. These genes were significantly enriched in functional categories related to immune response, inflammatory signaling, ERK1/ERK2 regulation, and macrophage differentiation (Figure 2B). These enrichment results indicate that the M2 polarization-related gene set is associated with immune- and macrophage-related programs and may reflect biological processes relevant to tumor progression.

To screen for genes associated with prognosis in patients with CRC, we first performed a univariant Cox regression analysis on the 311 M2 polarization-related genes. As a result, we found 32 genes that significantly regulated the prognosis of CRC. (Figure 2C) We next performed a LASSO analysis on these 32 genes and finally got 16 M2-associated prognostic genes (M2Gs), which were *CLEC11A*, *CTSD*, *DCTPP1*, *DNASE1L3*, *DSC2*, *FABP3*, *FABP5*, *FCGBP*, *GSTM2*, *HYI*, *ITM2C*, *NPL*, *PLTP*, *S100A13*, *SDS*, and *TUBB* (Figures 2D and 2E).

To validate reproducibility, we repeated the analysis using another scRNA-seq dataset (GSE231559). Again, macrophages were classified into M0-like, M1-like, and M2-like subsets (Figures S2A–S2D), and trajectory analysis identified 416 M2-related genes, of which 255 overlapped with the initial dataset, including all 16 M2Gs (Figures S3A–S3D). These findings highlight the conserved role of the 16 M2Gs in M2 polarization across CRC datasets.

### Validation of M2-specific genes and functional impact on macrophage polarization and tumor cells

**Figure 3:**
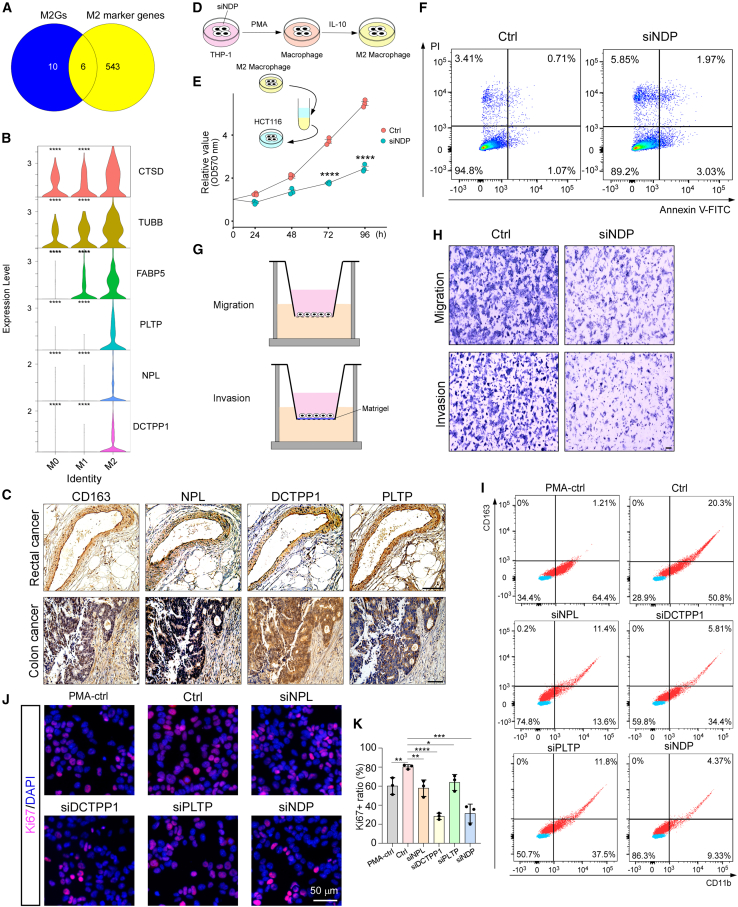
NPL, DCTPP1, and PLTP significantly affected M2 macrophage polarization and CRC cells tumorigenesis (A) Overlap between M2Gs and M2 macrophage marker genes. (B) Relative expression of *CTSD*, *TUBB*, *FABP5*, *PLTP*, *NPL*, and *DCTPP1* in M0-like, M1-like, and M2-like macrophages. (C) Immunohistochemical staining of CD163, PLTP, NPL, and DCTPP1 in colorectal cancer tissues, *n* = 8. (D) Schematic of THP-1-derived M2-like macrophage polarization. (E and F) Proliferation and apoptosis of HCT116 cells cultured with conditioned medium from Ctrl or siNDP macrophages, *n* = 3. (G and H) Schematic and representative images of Transwell migration and Matrigel invasion assays. (I) Representative flow cytometry plots of CD11b and CD163 expression in macrophages under the indicated treatments, *n* = 3. (J and K) Ki67 immunofluorescence and quantification in HCT116 cells cultured with conditioned media from the indicated groups, *n* = 3. Ctrl, control siRNA; PMA-ctrl, PMA-differentiated macrophages without IL-10; siNDP, equal-molar mixture of siRNAs targeting NPL, DCTPP1, and PLTP. ∗*p* < 0.05, ∗∗*p* < 0.01, ∗∗∗*p* < 0.001, and ∗∗∗∗*p* < 0.0001. Scale bars, 100 μm unless otherwise indicated.

To focus on genes highly expressed in M2-like macrophages, we screened the 16 M2Gs and found *CTSD*, *TUBB*, *FABP5*, *PLTP*, *NPL*, and *DCTPP1* to be consistently enriched, with *PLTP*, *NPL*, and *DCTPP1* exclusively expressed in M2-like cells across both datasets (Figures 3A, 3B, and S3E). Immunohistochemistry of CRC tissues further confirmed that these six genes colocalized with CD163^+^ M2 macrophages in both colon and rectal cancers (Figures 3C and S4A).

To assess their functional roles, we transfected THP-1 cells with siRNAs targeting *NPL*, *DCTPP1*, and *PLTP* (siNDP) and induced macrophage differentiation (Figure 3D). Conditioned media from siNDP-treated macrophages suppressed proliferation and migration while enhancing apoptosis of HCT116 CRC cells (Figures 3E–3H). These data indicate that NPL, DCTPP1, and PLTP facilitate M2 polarization and indirectly promote CRC cell tumorigenicity.

To further delineate the contribution of each gene individually, we silenced *NPL*, *DCTPP1*, or *PLTP* separately during macrophage differentiation, using PMA-treated cells without IL-10 as the M0-like baseline control (PMA-ctrl) (Figure 3I). As expected, IL-10 stimulation markedly increased the proportion of CD11b^+^CD163^+^ macrophages, consistent with the induction of an M2-like phenotype. Notably, knockdown of each gene significantly reduced the CD11b^+^CD163^+^ fraction compared with the IL-10 control (Ctrl group), with DCTPP1 silencing producing the most pronounced decrease. We observed a similar trend in U937-derived macrophages, further supporting the role of these genes in modulating IL-10–driven M2-like polarization, although the differential dominance of DCTPP1 was less evident in this model (Figure S4B). Consistently, Ki67 immunostaining of HCT116 cells cultured with macrophage-conditioned media further confirmed that these genes influence CRC cell behavior in a polarization-dependent manner (Figures 3J and 3K). Conditioned media from macrophages with NPL, DCTPP1, or PLTP knockdown attenuated the Ki67+ ratio relative to the IL-10 control, indicating reduced proliferative activity of CRC cells secondary to impaired M2-like polarization.

### Construction of an M2G-based prognostic risk model in CRC

**Figure 4:**
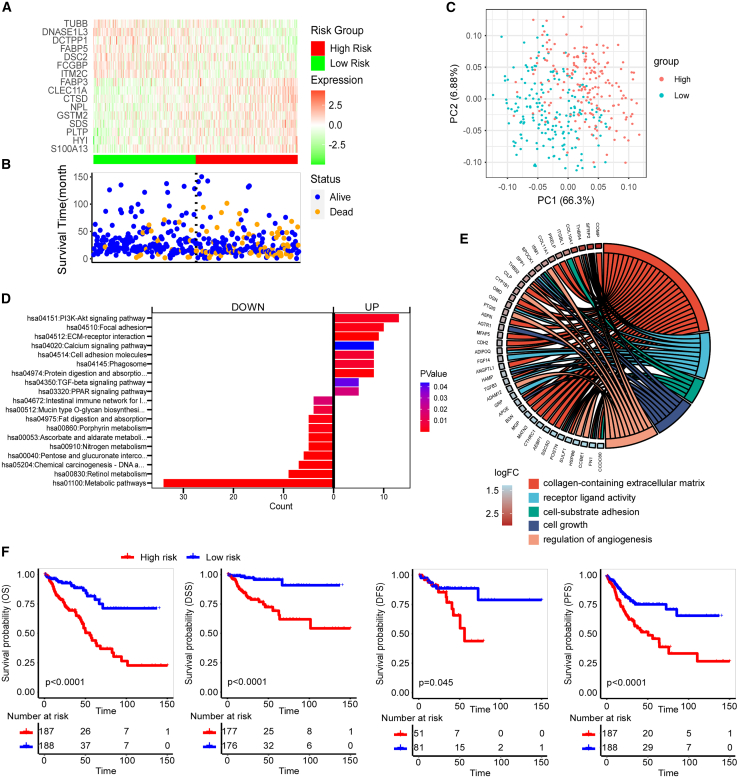
Relationship between the risk scores built with the 16 M2Gs and CRC prognosis (A) The relative expressions of the 16 M2Gs in the high- and low-risk groups. (B) The high-risk group displayed relatively more deaths and shorter survival times. (C) The PCA analysis showed that the high- and low-risk groups were well separated. (D) The altered KEGG pathways and (E) upregulated GO terms in the high- and low-risk groups. (F) The Kaplan-Meier survival curves of the high- and low-risk groups for OS, DSS, DFS, and PFS in CRC.

To investigate the prognostic implications of the 16 M2Gs, we built a risk model using their expression profiles. We first decided the relative expressions of the 16 M2Gs in the high- and low-risk groups, respectively. *SDS*, *GSTM2*, *S100A13*, *HYI*, *CLEC11A*, *NPL*, *FABP3*, *CTSD*, and *PLTP* were upregulated in the high-risk group, while the rest were downregulated. (Figure 4A) Moreover, the high-risk group displayed relatively more deaths and shorter survival times (Figure 4B and Table 1).

**Table 1:** Clinical characteristics of the TCGA cohort

	Risk_High (*N* = 187)	Risk_Low (*N* = 188)	*p*-value
SITE			0.743
Colon	142 (75.9%)	139 (73.9%)	
Rectum	45 (24.1%)	49 (26.1%)	
GENDER			0.0425
MALE	113 (60.4%)	93 (49.5%)	
FEMALE	74 (39.6%)	95 (50.5%)	
T	0 (0%)	1 (0.5%)	0.00138
TIS	2 (1.1%)	8 (4.3%)	
T1	16 (8.6%)	40 (21.3%)	
T2	139 (74.3%)	119 (63.3%)	
T3	28 (15.0%)	20 (10.6%)	
T4	2 (1.1%)	0 (0%)	
NA	0 (0%)	1 (0.5%)	
N			<0.001
NX	1 (0.5%)	1 (0.5%)	
N0	86 (46.0%)	118 (62.8%)	
N1	49 (26.2%)	50 (26.6%)	
N2	49 (26.2%)	19 (10.1%)	
NA	2 (1.1%)	0 (0%)	
M			0.0246
MX	29 (15.5%)	34 (18.1%)	
M0	119 (63.6%)	135 (71.8%)	
M1	33 (17.6%)	18 (9.6%)	
NA	6 (3.2%)	1 (0.5%)	
STAGE			<0.001
I	15 (8.0%)	41 (21.8%)	
II	66 (35.3%)	67 (35.6%)	
III	36 (19.3%)	41 (21.8%)	
IV	33 (17.6%)	19 (10.1%)	
NA	37 (19.8%)	20 (10.6%)	
AGE			0.717
≤65	90 (48.1%)	95 (50.5%)	
>65	97 (51.9%)	93 (49.5%)	
VITAL STATUS			<0.001
ALIVE	122 (65.2%)	167 (88.8%)	

TCGA, The Cancer Genome Atlas; NA. not applicable.

Subsequently, we mapped all TCGA samples to a PCA plot and found that the high- and low-risk groups were well separated. (Figure 4C) We determined the DEGs between the two groups and obtained 252 up-regulation genes and 196 down-regulation genes. We performed GO and KEGG analyses on the upregulated differentially expressed genes, revealing that these genes were significantly enriched in cell−substrate adhesion, cell growth, focal adhesion, and PI3K-AKT signaling pathway (Figures 4D and 4E). Notably, we further examined the effect of gene perturbation on AKT signaling in our functional assays. While combined knockdown of *NPL*, *DCTPP1*, and *PLTP* in macrophages resulted in an overall reduction of *p*-AKT levels, the magnitude of *p*-AKT changes induced by individual gene knockdown did not strictly mirror their respective effects on M2-like polarization and on CRC cell phenotypes observed in co-culture (Figure S4C). This discrepancy suggests that, although PI3K-AKT signaling is associated with the high-risk transcriptional program, these M2-associated genes may modulate macrophage polarization and CRC progression through additional pathways beyond AKT. Further mechanistic studies will be required to delineate the relative contribution of AKT-dependent versus AKT-independent mechanisms. We also plotted the Kaplan-Meier survival curves for OS, DSS, DFS, and PFS of the TCGA cohort according to the risk scores. In all these plots, the patients with higher risk scores displayed shorter survivals (Figure 4F).

### Validation of the prognostic model across independent CRC cohorts

**Figure 5:**
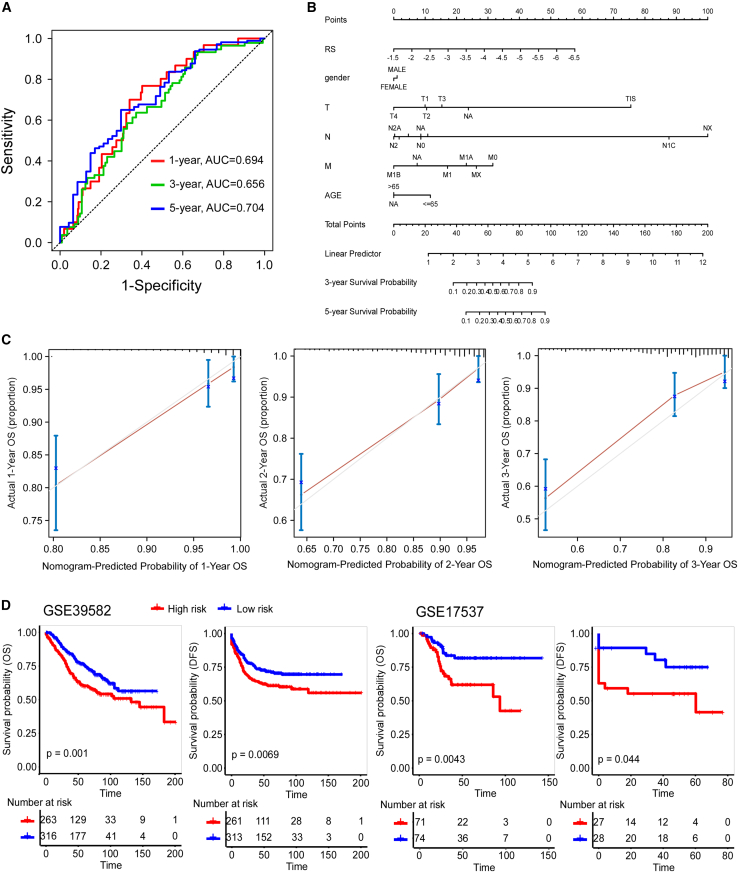
Prediction of prognosis in CRC based on the 16 M2Gs (A) The ROC analysis on the TCGA cohort with the 16 M2Gs. (B) The nomogram analysis with the risk scores calculated from the 16 M2Gs, TNM stages, and age to predict the OS of CRC. (C) The validation of the nomogram model using the actual 1, 2, and 3-year survival data. (D) The OS and DFS in the GSE39582 and GSE17537 cohorts were significantly shorter in the high-risk groups.

To evaluate the predictive value of 16 M2Gs identified in this study for the prognosis of CRC, we first performed an ROC analysis on the TCGA cohort with the 16 M2Gs. The areas under the ROC curve (AUCs) for 1, 3, 5, -year ROC curves were 0.694, 0.656, and 0.704, respectively (Figure 5A). Furthermore, we performed a nomogram analysis with the risk scores calculated from the 16 M2Gs, TNM stages, and age to predict the OS of CRC. As expected, higher risk scores suggested lower survival possibilities (Figure 5B). The predictive model from the nomogram analysis was also verified by the actual 1, 2, and 3-year survival data, respectively (Figure 5C).

To exclude the possibility that the risk score model built here only worked for the TCGA cohort, we examined it in two independent CRC cohorts (GSE39582 and GSE17537). The OS and DFS in both cohorts were significantly shorter in the high-risk groups, implying that the risk score model based on the 16 M2Gs could be widely used for the prediction of CRC prognosis (Figure 5D).

## Discussion

CRC is a disease of high recurrence and mortality. It is asymptomatic at early stages and thus usually diagnosed relatively late. Efficient and accurate strategies for CRC diagnosis and prognosis are in urgent need. Here, our integrative approach combining single-cell transcriptomics, bulk cohort validation, and functional assays provides insights into the immunological landscape of CRC. The identification of 16 conserved M2Gs underscores the robustness of macrophage polarization signatures across datasets. Beyond bioinformatic prediction, our siRNA experiments targeting *PLTP*, *NPL*, and *DCTPP1* in THP-1 derived macrophages revealed a causal role for these genes in M2 polarization. The consequent impairment of M2 polarization not only reduced the proportion of CD163^+^ macrophages but also curtailed CRC cell proliferation, migration, and invasion, while enhancing apoptosis in co-culture systems. These results strengthen the link between M2 macrophage enrichment and tumor-promoting phenotypes, suggesting that M2Gs are not merely prognostic biomarkers but also potential therapeutic targets for modulating the CRC immune microenvironment.

High-throughput sequencing can explore the pathogenesis of diseases at the molecular level, making it currently a powerful tool in the clinic. As to RNA sequencing, there are two types: single-cell and bulk RNA sequencing. Unlike the bulk one, single-cell RNA sequencing can detect gene expressions of every single cell and thus eliminate the interference of homogenization. In this study, we divide cells from CRC samples into different subgroups according to previously published marker genes, such as cancer cells, cancer stem cells, fibroblasts, endothelial cells, macrophages, and NK cells and so forth. Recent studies have revealed that macrophage polarization and M1/M2 ratio were associated with the survival of certain cancers.^19^ It is noted that M2 macrophages are usually recruited by tumors and secrete certain factors that promote tumorigenesis.^20^ Moreover, many references have documented the predictive values of macrophages in cancer prognosis.^21^^,^^22^ Therefore, we focused on macrophages in subsequent studies. By cell trajectory analysis, we found 311 M2 polarization-related genes. GO analysis showed that these genes mainly affect lymphocyte-mediated immunity, inflammatory response, positive regulation of immune response, and positive regulation of cell migration, which was consistent with the known function of M2 macrophages.^23^^,^^24^ KEGG analysis showed that these genes were involved in the PI3K-Akt signaling pathway, p53 signaling pathway, and NF-κB signaling pathway, which were typical pathways in tumorigenesis.^25^^,^^26^^,^^27^

Next, we performed a sequential univariate Cox regression analysis and LASSO analysis on the 311 M2 polarization-related genes and finally obtained 16 M2Gs, namely *CLEC11A*, *CTSD*, *DCTPP1*, *DNASE1L3*, *DSC2*, *FABP3*, *FABP5*, *FCGBP*, *GSTM2*, *HYI*, *ITM2C*, *NPL*, *PLTP*, *S100A13*, *SDS*, and *TUBB*. Among these, CTSD, FABP5, and TUBB have been reported to be upregulated in CRC, which usually leads to poor prognosis.^28^^,^^29^^,^^30^ Unlike this, high expressions of DNASE1L3, DSC2, FCGBP, and GSTM2 are beneficial for CRC prognosis.^31^^,^^32^^,^^33^^,^^34^ Moreover, CLEC11A has been identified as a prognostic gene in acute myeloid leukemia, while DCTPP1 affects the prognosis of prostate cancer.^35^^,^^36^ To further assess the robustness of these identified genes, we analyzed an independent CRC scRNA-seq dataset using the same workflow. Notably, all 16 M2Gs were retained among the overlapping M2 polarization-related genes identified in the validation dataset, supporting the reproducibility of their association with M2-like macrophage polarization.

The risk score is a widely used model in evaluating the effect of multiple genes on cancer prognosis.^37^ We here performed a multivariant Cox regression analysis on the 16 M2Gs and calculated the risk scores based on their expressions and coefficients. As shown in Table 1, the high-risk group was more prone to be advanced in both TNM and histological stages and had higher mortality. Interestingly, the percentage of high-risk patients was significantly higher in male patients than in females. This may be due to the differences in sex hormones and diet between males and females.^38^

The high-risk group tended to be of poor prognosis, as indicated by the Kaplan-Meier survival curves of OS, DSS, DFS, and PFS in CRC. Functional analysis of the DEGs between the high- and low-risk groups showed that the genes upregulated in the high-risk group were more associated with cell adhesion, growth, and angiogenesis, which favored tumorigenesis.^39^^,^^40^ However, the genes enriched in the low-risk group promoted receptor activator activity, metabolism, and immune responses, which were likely to be anti-tumor processes.^41^^,^^42^ We further evaluated the prognostic performance of the 16 M2G-based risk score in CRC. In addition to the TCGA cohort, the risk model was validated in two independent external datasets, in which it also stratified patients into groups with significantly different survival outcomes, including OS and DFS. ROC and nomogram analyses showed a modest but stable concordance between predicted and observed overall survival, indicating that the risk score may have adjunctive prognostic value rather than serving as a standalone clinical tool, while also underscoring the need for further validation before clinical application.

Beyond prognostic relevance, our functional perturbation experiments suggested that selected M2-associated genes may contribute to CRC progression by regulating both macrophage polarization and tumor-promoting signaling in cancer cells. Mechanistically, M2 macrophage polarization, particularly immunoregulatory programs induced by mediators such as IL-10, has been linked to the activation of the PI3K/AKT pathway and downstream signaling nodes (e.g., STAT3), which collectively support macrophage immunosuppressive functions and tumor-promoting activities.^43^ Our observation that knockdown of NPL, DCTPP1, and PLTP reduces *p*-AKT level provides an initial mechanistic connection between an M2-skewed macrophage program and oncogenic signaling in CRC, while acknowledging that macrophage states are plastic and likely integrate multiple pathways beyond AKT.

M2 macrophages are also likely to influence CRC progression through continuous crosstalk with the TME. Previous studies have shown that polarized TAMs secrete cytokines, chemokines, growth factors, and proteases that promote immunosuppression, angiogenesis, extracellular matrix remodeling, invasion, and metastasis.^44^ TAMs may also reinforce local immune suppression by recruiting or sustaining regulatory immune cell populations, thereby creating a microenvironment that favors tumor growth and dissemination.^45^ Consistent with this view, our conditioned medium experiments showed that macrophages with attenuated M2-like polarization lost part of their tumor-promoting effects on HCT116 cells, supporting a functional role for macrophage-tumor crosstalk in CRC progression.

Metabolic alterations in the TME may provide an additional explanation for these observations. Lactate accumulation is a well-recognized feature of solid tumors and has increasingly been implicated in the regulation of immune cell behavior, including the promotion of M2 macrophage polarization. In CRC, tumor-derived lactate has been reported to drive M2 polarization through TRAF6/IL-6/STAT3 signaling and to further promote tumor progression.^46^ Moreover, recent studies suggested that lactate can influence tumor progression not only as a metabolic byproduct but also through protein or histone lactylation, thereby linking metabolic reprogramming to epigenetic regulation, immune suppression, and poor prognosis.^47^ These findings provide additional context for understanding how M2-like TAM programs may contribute to aggressive tumor behavior and adverse clinical outcomes in CRC.

### Limitations of the study

This study has several limitations. First, the number of macrophages included for M2-associated gene discovery was relatively modest (611 cells), which may restrict the resolution for identifying transcriptional heterogeneity and specific subtypes. Second, the context-dependent nature of TAM polarization and tumor heterogeneity may limit the generalizability of the identified gene signatures across different tumor types or clinical settings. Third, although we conceptually frame macrophage activation using the classical M1/M2 paradigm and focus on M2-associated genes, macrophage polarization *in vivo* occurs along a continuum of intermediate states. Therefore, our signature likely captures a predominant M2-like axis of activation rather than the full spectrum of TAM phenotypes, and some functionally relevant states may not be fully represented. Fourth, although batch correction methods were applied, residual technical variation or inter-sample differences may still have influenced clustering results and marker selection. Future studies leveraging larger and more diverse single-cell datasets, as well as orthogonal validation techniques such as spatial transcriptomics or proteomics, will be essential to validate and refine the robustness of our findings.

## Resource availability

### Lead contact

Requests for further information and resources should be directed to and will be fulfilled by the lead contact, Lingling Dong (llingdong24@163.com).

### Materials availability

This study did not generate new unique reagents.

### Data and code availability

• This article does not report any newly generated datasets. The datasets used in this study were obtained from public databases, including GEO: GSE200997, GSE231559, GSE39582, GSE17537 and TCGA colorectal cancer datasets.
• This article does not report original code. The TCGA and GEO data codes are seen in the key resources table.
• Any additional information required to reanalyze the data reported in this article is available from the lead contact upon request.

## Acknowledgments

This work was supported by the 10.13039/501100010814Educational Commission of Anhui Province of China (no. 2022xscx038).

## Author contributions

Conceptualization, M.L. and L.D.; methodology, M.L. and L.D.; software, M.L. and L.D.; investigation, M.L. and L.D.; writing – original draft, M.L.; writing – review and editing, L.D.; visualization, M.L. and L.D.; supervision, L.D.; project administration, L.D.; funding acquisition, L.D. All authors contributed to the article and approved the submitted version.

## Declaration of interests

The authors declare no competing interests.

## Declaration of generative AI and AI-assisted technologies in the writing process

During the preparation of this work, the author(s) used DeepSeek in order to improve readability and language. After using this tool or service, the author(s) reviewed and edited the content as needed and take(s) full responsibility for the content of the publication.

## STAR★Methods

### Key resources table

**Table:** 

REAGENT or RESOURCE	SOURCE	IDENTIFIER
**Antibodies**		

CD163	Sangon Biotech	Cat# D160965; RRID: AB_3740921
Ki67	Proteintech	Cat# 28074-1-AP; RRID: AB_2918145
NPL	Sangon Biotech	Cat# D123828; RRID: AB_3740922
DCTPP1	Sangon Biotech	Cat# D126081; RRID: AB_3740923
PLTP	Sangon Biotech	Cat# D122892; RRID: AB_3740924
CTSD	Sangon Biotech	Cat# D220354; RRID: AB_3740925
TUBB	Sangon Biotech	Cat# D223070; RRID: AB_3740926
FABP5	ABclonal	Cat# A27255; RRID: AB_3740927
*p*-Akt (S473)	Invitrogen	Cat# MA1-20325; RRID: AB_557538
GAPDH	Biosharp	Cat# BL006B; RRID: AB_2890028
CD11b-conjugated APC antibody	Proteintech	Cat# APC-65116; RRID: AB_2883004
CD163-conjugated FITC antibody	Proteintech	Cat# FITC-65561; RRID: AB_3740928
Donkey anti-Rabbit HRP secondary antibody	Invitrogen	Cat# 31458; RRID: AB_228213
Goat anti-Mouse HRP secondary antibody	Biosharp	Cat# BL001A; RRID: AB_2827665
Goat anti-mouse Alexa Fluor Plus 555	Invitrogen	Cat# A32727; RRID: AB_2633276

**Chemicals, peptides, and recombinant proteins**		

RPMI 1640	Gibco	Cat# 11875093
L-Glutamine	Gibco	Cat# 25030081
Fetal Bovine Serum	Clark	Cat# FB15015
2-Mercaptoethanol	Merck	Cat# M6250
DMEM	HyClone	Cat# SH30022.01
Phorbol 12-myristate 13-acetate (PMA)	Merck	Cat# P8139
recombinant human interleukin-10 (IL-10)	MCE	Cat# HY-P7030
Matrigel	Corning	Cat# 356234

**Critical commercial assays**		

BCA Protein Assay Kit	Biosharp	Cat# BL521A
Lipofectamine™ RNAiMAX	Invitrogen	Cat# 13778100
MTT assay kit	Sangon Biotech	Cat# E606334
Annexin V/PI apoptosis detection kit	Sangon Biotech	Cat# E606336
Immunohistochemical Kit	Sangon Biotech	Cat# D601037

**Deposited data**		

CRC scRNA-seq dataset	GEO database	GSE200997, GSE231559
CRC bulk RNA-seq dataset	UCSC Xena database, GEO database	https://xenabrowser.net/datapages/, GSE39582, GSE17537

**Experimental models: Cell lines**		

THP-1	CAS Cell Bank	N/A
HCT116	CAS Cell Bank	N/A
U-937	CAS Cell Bank	N/A

**Oligonucleotides**		

Negative Control	MCE	Cat# HY-150150
siNPL-Sense: GGGCUGAAAUUCAGUGAUATT; siNPL-Antisense: UAUCACUGAAUUUCAGCCCTT	This paper	N/A
siDCTPP1-Sense: CAUCAACCGGCGACGCUACTT; siDCTPP1-Antisense: GUAGCGUCGCCGGUUGAUGTT	This paper	N/A
siPLTP-Sense: GGUUCCGAAUCUAUUCCAATT; siPLTP-Antisense: UUGGAAUAGAUUCGGAACCTT	This paper	N/A

**Software and algorithms**		

GraphPad Prism10	GraphPadSoftware	https://www.graphpad.com
FlowJo 10.8.1	Treestar	https://www.flowjo.com/solutions/flowjo
RStudio	RStudio Software	https://posit.co/download/rstudio-desktop/
R version 4.5.0	R Core Team	https://www.R-project.org/
ImageJ	ImageJ Software	https://imagej.nih.gov/

### Experimental model and study participant details

#### Tumor sample tissues

All human tissue samples used for immunohistochemistry (IHC) were collected with written informed consent from all participants, in accordance with the Declaration of Helsinki and under protocols approved by the Ethics Committee of Anhui Normal University (Approval No. AHNU-ET2022032). All samples were anonymized prior to analysis, and detailed information is shown in Table S4.

#### Cell lines

THP-1, U-937 and HCT116 cells were kindly provided by the Stem Cell Bank, Chinese Academy of Sciences. THP-1 and U-937 cells were maintained in RPMI 1640 (Gibco) medium supplemented with 10% FBS (Clark) and 0.05 mM 2-Mercaptoethanol. HCT116 cells were cultured in DMEM (HyClone) supplemented with 10% FBS. Cultures were incubated at 37 °C in a humidified atmosphere containing 5% CO_2_. Cells were routinely screened for mycoplasma contamination and only mycoplasma-negative cultures were used for experiments.

### Method details

#### Cell culture

To induce M2 polarization, THP-1 and U-937 cells were seeded into tissue-culture-treated 6-well plates at 5x 10ˆ5 cells/well and allowed to equilibrate for 16–24 h. Cells were then treated with phorbol 12-myristate 13-acetate (PMA) (Sigma) at a final concentration of 5 ng/mL for 24 h to promote adherence and differentiation toward a macrophage-like phenotype. After 24 h, PMA-containing medium was removed, wells were gently washed twice with prewarmed PBS to remove residual PMA, and PMA-free medium was added. Cells were allowed to recover for 72 h to stabilize the M0 phenotype and minimize acute PMA effects. Following recovery, cells were exposed to recombinant human interleukin-10 (IL-10) (MCE) at 20 ng/mL for 48 h to induce M2-like polarization.

#### MTT assay

Cell viability and proliferation were assessed using an MTT assay kit (Sangon) according to the manufacturer’s instructions. HCT116 cells were exposed to THP-1-derived conditional medium from different treatment groups for defined intervals, and metabolic activity was quantified by measuring the conversion of MTT to formazan by viable cells. Briefly, Cells were seeded into 96-well plates at a density of 2,000 cells per well. Cell viability was assessed at 0, 24, 48, 72, and 96 h post-seeding. At each time point, MTT reagent was added, and the formazan product was measured by absorbance at 570 nm using a TECAN microplate reader. Absorbance values at each time point were normalized to the 0 h measurement to account for initial cell seeding differences.

#### Flow cytometry

Cultured cells were trypsinized to obtain a single-cell suspension and briefly washed with PBS. Cells were then incubated with human Fc receptor blocking reagent for 20 min at room temperature to reduce nonspecific binding. Following blocking, cells were stained with the CD11b-conjugated APC antibody (Proteintech) and CD163-conjugated FITC antibody (Proteintech) for 30 min at 4 °C in the dark. After washing with PBS, samples were analyzed on a BD FACSCanto II flow cytometer (BD Biosciences).

For apoptosis, cells were assessed using an Annexin V/PI apoptosis detection kit (Sangon) following the manufacturer’s instructions. Briefly, cells were trypsinized to a single-cell suspension and resuspended in the provided binding buffer. Cells were then sequentially incubated with Annexin V-FITC for 15 min and propidium iodide (PI) for 30 min in the dark at room temperature. Samples were immediately analyzed by flow cytometry.

#### Cell migration and invasion assay

Cell migration was assessed using Transwell inserts (Corning). For migration assays, 4.0x 10ˆ4 CRC cells in serum-free medium were seeded into the upper chamber, and medium containing 20% FBS supplemented with THP-1 conditioned medium from the respective treatment groups was placed in the lower chamber as a chemoattractant. For invasion assays, the upper surface of the insert membrane was coated with Matrigel (BD Biosciences) diluted at a ratio of 1:8. For invasion assays, 9.0x 10ˆ4 cells in serum-free medium were seeded into the Matrigel-coated upper chamber, and the lower chamber contained the same 20% FBS medium supplemented with the corresponding THP-1 conditioned medium.

After incubation at 37 °C in a humidified atmosphere with 5% CO2 for 48 h, non-migrated/non-invaded cells on the upper surface of the membrane were removed by gentle swabbing. Membranes were washed with prewarmed calcium-free PBS, fixed in methanol for 30 min, and stained with 0.1% crystal violet for 20 min. Migrated/invaded cells on the lower surface of the membrane were imaged and counted under an inverted microscope.

#### Immunostaining

For cultured cells, samples were briefly rinsed with PBS and fixed with 4% paraformaldehyde (PFA) for 10 min at room temperature. Cells were then washed with PBS and permeabilized with 0.1% PBST for 30 min. Subsequently, cells were blocked with blocking buffer (10% normal goat serum and 0.1% Triton X-100 in PBS) at 37 °C for 1 h in a humidified chamber. Primary antibodies (anti-Ki67, 1:200, Proteintech, Cat No. 28074-1-AP) diluted in blocking buffer were applied and incubated overnight at 4 °C. On the following day, cells were incubated with secondary antibodies (Goat anti-mouse Alexa Fluor Plus 555-conjugated IgG, 1:500, Invitrogen, Cat No. A32727) diluted in PBS for 90 min at room temperature. After two washes with 0.1% PBST, nuclei were counterstained with DAPI for 5 min. Cells were kept in PBS and imaged immediately. Images were acquired using a Leica DMi8 microscope.

#### Western blot

Proteins were extracted using lysis buffer supplemented with a protease inhibitor cocktail and a phosphatase inhibitor cocktail. Protein concentrations were measured using a BCA Protein Assay Kit (Biosharp, Cat. No. BL521A), and equal amounts of protein were loaded for each sample. Proteins were separated by SDS-PAGE and transferred onto polyvinylidene fluoride (PVDF) membranes. Membranes were blocked with 5% (w/v) nonfat milk for 1 h at room temperature and then incubated with primary antibodies (anti-*p*-Akt (S473) mAb, 1:1000, Invitrogen, Cat No. MA1-20325; anti-GAPDH pAb, 1:2000, Biosharp, Cat No. BL006B) overnight at 4 °C. After washing, membranes were incubated with the appropriate secondary antibodies (goat anti-mouse HRP secondary antibody, 1:2000, Biosharp, Cat No. BL001A) for 90 min at room temperature. Protein bands were visualized using a chemiluminescent substrate, and signals were captured using a Tanon 5200 imaging system.

#### Dimension reduction and annotation of scRNA-seq data

scRNA-seq data were first analyzed using the Seurat package in R. The genes that were expressed in fewer than 3 cells and cells expressing fewer than 200 genes or with >25% of total UMI counts derived from mitochondrial genes were filtered out. The data were further subjected to doublet filtering using the DoubletFinder package in R, with default parameter settings for the expected doublet rate. After these quality control steps, 28,257 cells and 23,032 genes remained for downstream analysis. Subsequently, the top 2,000 variable genes were selected using the variance stabilizing transformation (VST) method in Seurat, and canonical correlation analysis (CCA) was applied to correct batch effects across samples. Cell-cycle effects were mitigated by computing S-phase and G2/M-phase scores from canonical marker genes and regressing these scores out during data scaling. Next, we determined the top 2000 variable genes and performed a principal component analysis (PCA) on the scRNA-seq data. Subsequently, we run a Uniform Manifold Approximation and Projection (UMAP) analysis using the top 30 principal components from the PCA. With resolution = 0.5, we divided all cells into 23 clusters. According to canonical cell markers, we finally annotated 12 cell subgroups, namely Cancer stem cell, Fibroblast, Cancer cell, CD8^+^ T cell, Plasma, Macrophage, Endothelial cell, Treg, Th cell, NK cell, Mast cell, and B cell. These marker genes used for cell cluster annotation were listed in Table S1.

#### Cell trajectory analysis of scRNA-seq data

We extracted the macrophage part from the scRNA-seq data for further investigations. With resolution = 1.0, we divided macrophages into 7 clusters. As the CellMarker database and references suggested, we annotated M1-like macrophages with *TNF*, *IL1B*, and *ITGAX* markers and M2-like macrophages with *MRC1*, *CD163*, *LGMN*, and *MMP12* markers,^48^^,^^49^^,^^50^^,^^51^^,^^52^^,^^53^^,^^54^ among which *TNF* and *IL1B* (as M1 markers) and *MRC1* and *CD163* (as M2 markers) have been reported as robust M1/M2-like macrophage markers in colorectal cancer.^55^^,^^56^^,^^57^^,^^58^ The rest cells were termed M0-like macrophages. To further validate these annotations and avoid reliance on a narrow marker panel, we compiled extended M1 and M2 signature gene sets from the literature and calculated module scores for each cell.^59^^,^^60^^,^^61^^,^^62^^,^^63^ Consistent with our marker-based classification, the M1-like cluster exhibited significantly higher M1 signature scores, whereas the M2-like cluster showed significantly higher M2 signature scores, with M0-like macrophages displaying comparatively lower scores for both signatures. The full gene lists were provided in Table S2. To identify specific marker genes of each macrophage subtype, we used the “FindMarkers” function from the Seurat package to screen for genes in the macrophage subgroup, with the default parameters and *p* value <0.05 as a significance cutoff. Finally, the 549 marker genes of M2 macrophages were listed in Table S5. Next, we performed pseudotime trajectory analysis using the Monocle2 package in R and identified 311 M2 polarization-related genes based on differential expression along pseudotime with a stringent cutoff (q value <1 × 10^−9^). The 311 M2 polarization-related genes were listed in Table S6.

#### Cox regression and least absolute shrinkage and selection operator (LASSO) analysis

We performed sequential univariant Cox and LASSO analyses on the 311 M2 polarization-related genes to identify the genes significantly associated with the prognosis of CRC. Univariant Cox and LASSO analyses were performed with the survival and glment packages in R, respectively. In the univariate Cox regression step, genes with *p* < 0.05 and satisfying the proportional hazards assumption (Schoenfeld test *p* > 0.05) were retained. To further reduce false positives and improve model stability, the candidate genes were subjected to LASSO Cox regression with 10-fold cross-validation and 1,000 bootstrap iterations. Only genes selected in ≥85% of iterations were included in the final prognostic model. Finally, we identified 16 M2Gs, which were *CLEC11A*, *CTSD*, *DCTPP1*, *DNASE1L3*, *DSC2*, *FABP3*, *FABP5*, *FCGBP*, *GSTM2*, *HYI*, *ITM2C*, *NPL*, *PLTP*, *S100A13*, *SDS*, *TUBB* for further studies. We performed a multivariant Cox regression analysis on the 16 M2Gs and calculated the risk score as follows:

$\text{Risk}\;\text{Scores}\;\left(\text{RS}\right)=\sum_{1}^{n}\mathrm{Exp}\;\left(GENEn\right)*\beta n$, in which *Exp (GENEn)* represents the gene expression levels and *βn* represents the corresponding regression coefficients. The regression coefficients of 16 M2Gs were listed in Table S3. Based on the median risk scores, we divided the patients in each cohort into high- and low-risk groups. The differences in clinical parameters between the high- and low-risk groups in the TCGA cohort were shown in Table 1.

#### Bulk RNA-seq data analysis

RNA-seq gene expression and corresponding clinical data for colorectal cancer (COAD and READ) were obtained from the UCSC Xena platform (https://xenabrowser.net), using the dataset “TCGA COADREAD gene expression by RNAseq (polyA + IlluminaHiSeq)”. This dataset contains RSEM-normalized expression values provided in log_2_(x + 1) format. For downstream modeling analyses, we approximated pseudo-count values by applying an inverse transformation using the formula expression = 2ˆ(log2_RSEM) - 1. This approach allowed us to restore relative expression magnitudes suitable for regression-based modeling (e.g., univariate Cox and LASSO analyses). Differentially expressed genes were analyzed using the DESeq2 package in R. The adjusted *p* value ≤ 0.05 and |log2(FoldChange)| > 1 were used as the cutoff. The Gene Ontology (GO) was performed using the clusterProfiler package and visualized using the GOplot package in R. The Kyoto Encyclopedia of Genes and Genomes (KEGG) analysis was performed using The Database for Annotation, Visualization and Integrated Discovery (DAVID) platform (https://david.ncifcrf.gov/) and visualized using the ggplot2 package in R.

For external validation, raw microarray data (GSE39582 and GSE17537) were preprocessed using the robust multi-array average (RMA) algorithm implemented in the affy packages, including background correction, quantile normalization, and log2 transformation. Probe sets were mapped to official gene symbols according to the corresponding platform annotation, and when multiple probe sets mapped to the same gene, their median expression value was used.

#### Kaplan-Meier survival analysis

For each cohort (TCGA, GSE39582, and GSE17537), expression values of the 16 M2Gs were first standardized using a gene-wise *Z* score transformation. The risk score of each patient was then calculated based on the standardized expression of the 16 M2Gs and the corresponding regression coefficients derived from the TCGA cohort. Patients in each cohort were equally divided into high- and low-risk groups according to the cohort-specific median risk score. OS, DSS, DFS, and PFS were subjected to Kaplan–Meier analyses, and *p* < 0.05 was considered statistically significant.

#### Receiver operating characteristic and nomogram analyses

The expressions of the 16 M2Gs were used to predict the prognosis of CRC and the prediction accuracy was evaluated by the Receiver Operating Characteristic (ROC) and nomogram analyses. The ROC and nomogram analyses were performed using the timeROC and RMS packages in R, respectively. The overall survival data in the TCGA cohort were used for validations in the ROC and nomogram analyses.

#### Immunohistochemical staining

The 8 colon cancer and 8 rectal cancer patient samples used in this study. We paraffin-embedded colon and rectal cancer tissues and performed immunohistochemical staining following tissue sections using an Immunohistochemical Kit (Sangon Biotech, Cat# D601037) according to the manufacturer’s instructions. Briefly, the sample slides were subjected to wax removal by xylene and rehydrated with gradient ethanol. Antigen retrievals were performed with the boiling citrate solution. The sample slides were blocked with 3% bovine serum albumin and then incubated with primary antibodies overnight at 4°. On the second day, the slides were incubated with HRP-conjugated secondary antibodies for 30 min, followed by color reaction using the DAB method. Nucleus was stained with hematoxylin dye solution. Finally, the slices were sealed with neutral gum after gradient dehydration and xylene treatment. The staining results were observed with a Leica DMi8 fluorescence microscope.

### Quantification and statistical analysis

#### Statistical analysis

All statistical analyses were performed using R and GraphPad Prism. Flow cytometry data were analyzed using FlowJo, and image quantification was performed using ImageJ. Unless otherwise indicated, data are presented as mean ± standard deviation (SD) from three independent biological replicates. The exact value of n and the definition of n for each experiment are provided in the corresponding figure legends. Statistical details, including the statistical tests used, sample sizes, and significance information, can be found in the figure legends and relevant Results sections.For comparisons between two groups, two-sided Student’s t-tests were used. For comparisons among three or more groups, one-way analysis of variance (ANOVA) was applied. A *p* value <0.05 was considered statistically significant.

## Supplemental information

Document S1. Figures S1–S4, Tables S1–S4 and Data S1

Table S5. 549 M2 macrophage marker genes

Table S6. 311 M2 polarization-related genes
