# Supplementary material for: M2 macrophage associated genes shape prognosis and tumor progression in human colorectal cancer
Source: iScience. 2026 Jun 5;29(6):116230. doi: 10.1016/j.isci.2026.116230 (PMC13264372; doi:10.1016/j.isci.2026.116230)
Supplement: Document S1. Figures S1–S4, Tables S1–S4 and Data S1 [file mmc1.pdf]

iScience, Volume *29*

Supplemental information

M2 macrophage associated genes shape prognosis and tumor progression in human colorectal cancer

Meng Li and Lingling Dong

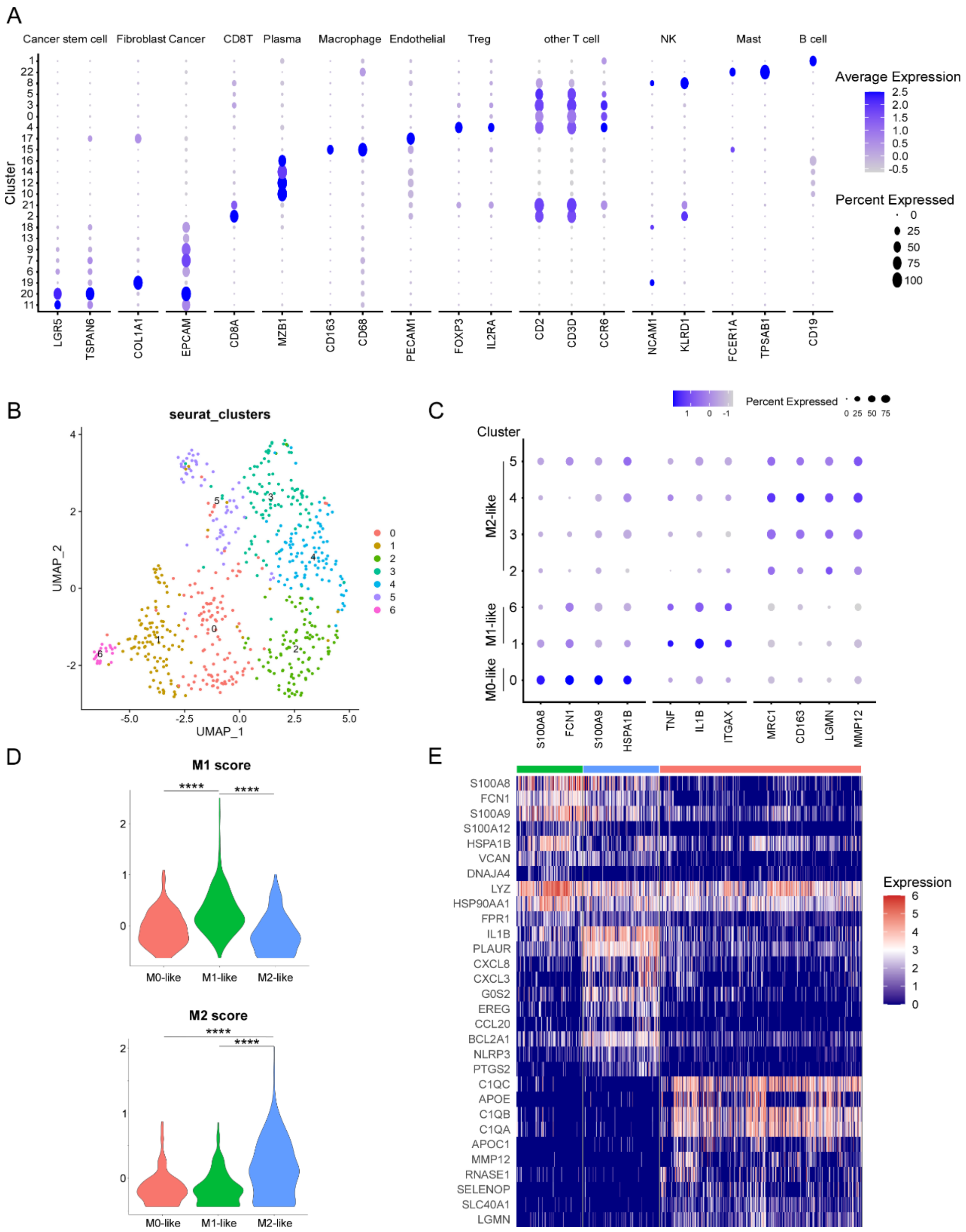

Figure. S1 Using cell markers annotated different clusters of scRNA-seq data. (**A**) The dot plot showing that 23 clusters were annotated into 12 cell types according to specific cell markers. (**B**) The UMAP plot of all 7 clusters of macrophages the scRNA-seq data. (**C**) The dotplot showing that 7 clusters of macrophages were annotated into M0-like, M1-like, and M2-like macrophages according to M0, M1, and M2 cell

markers. (**D**) Violin plots showing the M1 (upper) and M2 (lower) module scores across M0-like, M1-like, and M2-like macrophages. Module scores were calculated using Seurat (AddModuleScore) based on extended M1/M2 signature gene sets. ****, $P < 0.0001$. (**E**) The heatmap showed genes specifically expressed in each macrophage subtype.

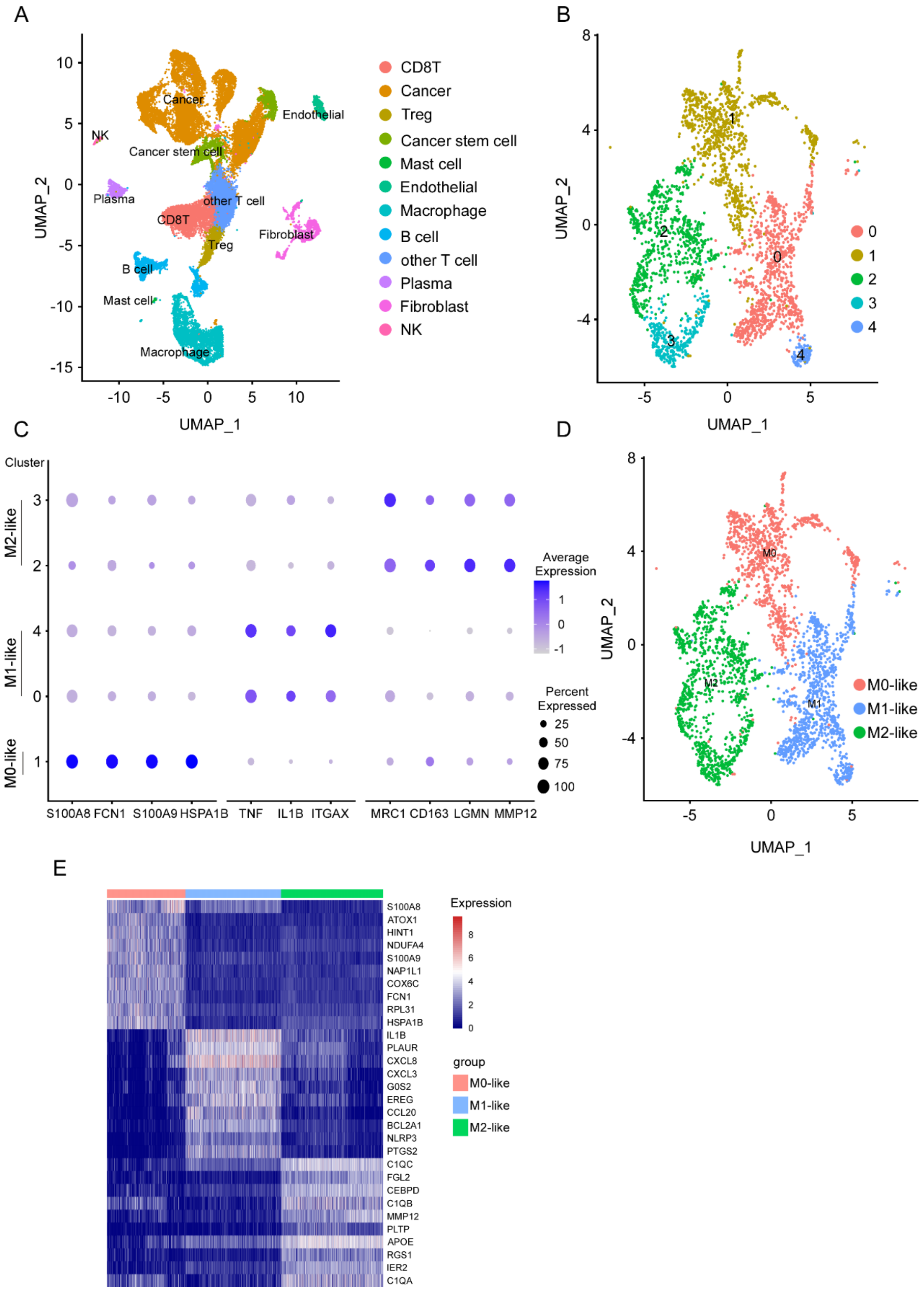

Figure. S2 Macrophage subgroup annotation and marker identification in another CRC scRNA dataset, GSE231559. (**A**) The UMAP plot of the GSE231559 dataset, with the macrophage subgroup was shown in (**B**). The (**C**) Dotplot and (**D**) UMAP plot

showing the annotation of each macrophage subtype. (**E**) The heatmap showing the specific expression genes of each macrophage subtype.

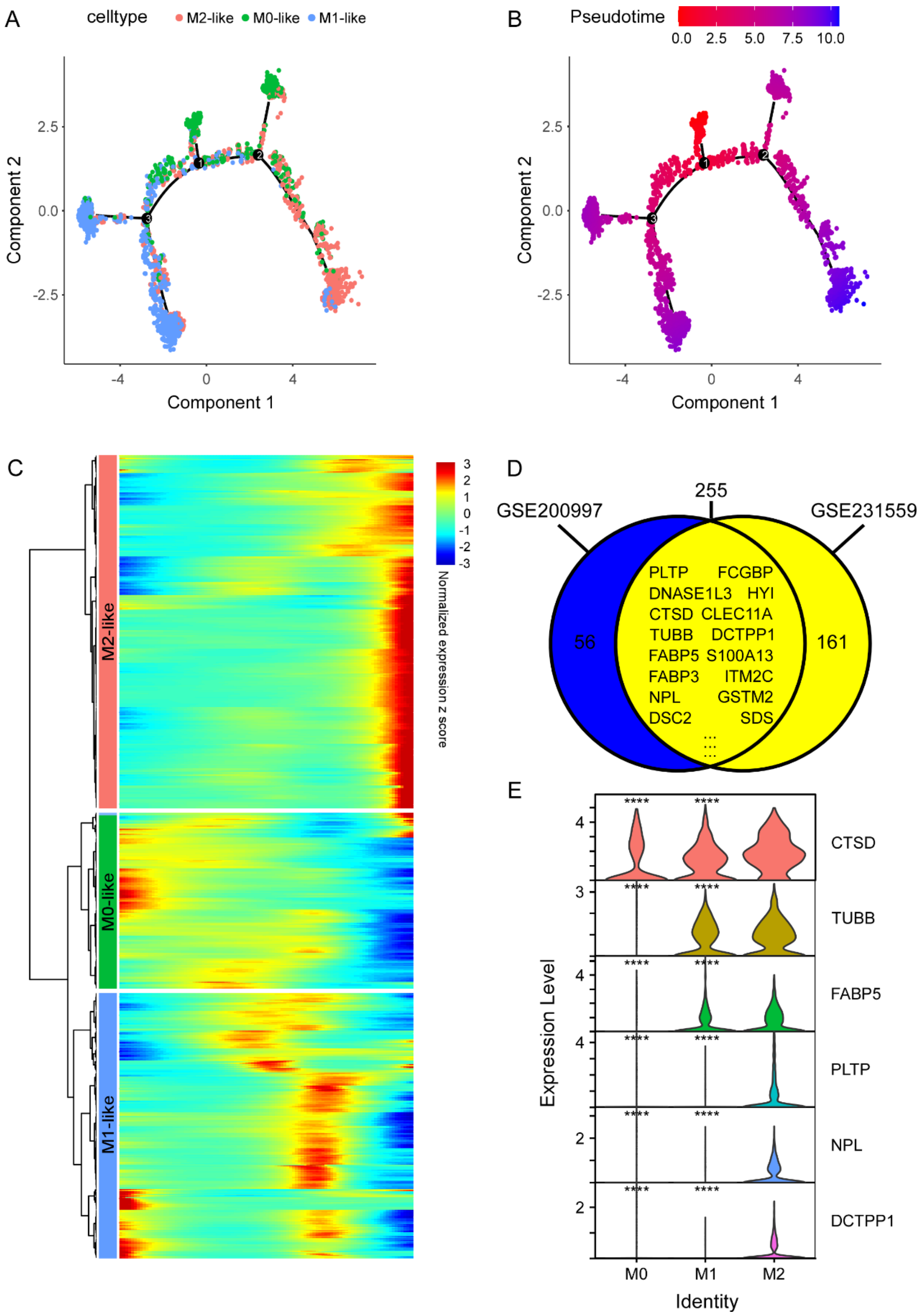

Figure. S3 Validation of 16 M2Gs in GSE231559 dataset. (**A**) The macrophage polarization trajectory was plotted based on both cell types and (**B**) pseudotime values,

respectively. (**C**) The heatmap displayed expression patterns closely correlated with the cell trajectory. (**D**) The Venn diagram shows 255 overlapping genes in the GSE200997 and GSE231559 datasets, including the 16 M2Gs. (**E**) The relative expressions of *CTSD*, *TUBB*, *FABP5*, *PLTP*, *NPL*, and *DCTPP1* in M0-like, M1-like, and M2-like macrophages of the GSE231559 dataset, respectively. In addition, the gene expressions in the M0-like and M1-like macrophages were compared to the M2-like macrophages, respectively, with asterisk labels represents the significance extents. ****，P value < 0.0001.

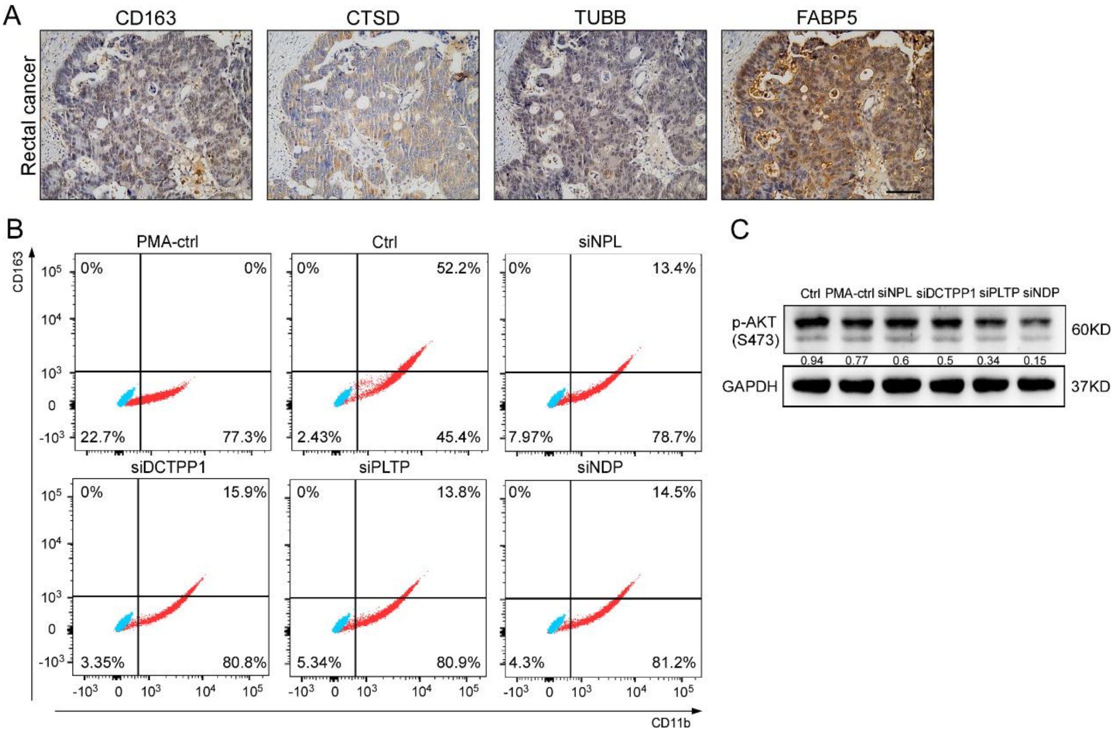

Figure. S4 Validation of candidate macrophage markers and effects of NPL/DCTPP1/PLTP knockdown on M2-like polarization and AKT signaling. (**A**) Representative immunohistochemical staining of CD163, CTSD, TUBB, and FABP5 in rectal cancer tissue sections. n=8, Scale bar, 100 μm. (**B**) Validation in U937-derived macrophages. Flow cytometric analysis of CD11b and CD163 expression under the indicated conditions (PMA-ctrl, Ctrl, siNPL, siDCTPP1, siPLTP, and siNDP). The percentages shown indicate the fraction of cells in each quadrant, n = 3. (**C**) Western blot analysis of p-AKT (Ser473) and GAPDH in HCT116 cells treated with conditioned media from macrophages under the indicated conditions. Numbers denote the relative band intensity (normalized to GAPDH), n = 3. Ctrl, control siRNA; PMA-ctrl, PMA-differentiated macrophages without IL-10; siNDP, mixtures of siRNA for NPL, DCTPP1 and PLTP at equal molar ratios.

Methods S1: Supplementary tables supporting the Methods section

Table S1. Marker genes used for cell cluster annotation

**Cell types**	**Marker genes**
Cancer stem cell	*LGR5*, *TSPAN6*
Fibroblast	*COL1A1*
Cancer	*EPCAM*
CD8T	*CD8A*
Plasma	*MZB1*
Macrophage	*CD163*, *CD68*
Endothelial	*PECAM1*
Treg	*FOXP3*, *IL2RA*
other T cell	*CD2*, *CD3D*, *CCR6*
NK	*NCAM1*, *KLRD1*
Mast	*FCER1A*, *TPSAB1*
B cell	*CD19*

Table S2. Extended M1 and M2 macrophage signature gene sets

**Types**	**Genes**
M1 signature	*"IL1B","TNF","CXCL9","CXCL10","CXCL11",*<br>*"CCR7","CD80","CD86","STAT1","IRF1","NFKBIA","ITGAX"*
M2 signature	*"MRC1","CD163","MS4A4A","CCL18","CCL17",*<br>*"CCL22","TREM2","MERTK","TGFB1",*<br>*"IL10","MMP9","VEGFA","LGMN","MMP12"*

Table S3. The Beta-regression coefficients of 16 M2Gs

Genes	Beta-regression coefficients
PLTP	0.130714466
DNASE1L3	-0.147660985
CTSD	0.072405632
TUBB	-0.544133099
FABP5	-0.11808276
FABP3	0.003074181
NPL	0.099250778
FCGBP	-0.034957855
CLEC11A	0.04234698
DCTPP1	-0.138728476
HYI	0.18428086
S100A13	0.219478774
ITM2C	-0.026169392
GSTM2	0.107314468
DSC2	-0.076822481
SDS	0.118850414

Table S4. Patients information

**Sample Numble**	**Age**	**Sex**	**Pathology Diagnosis**
CC01	62	Male	Adenocarcinoma
CC02	71	Female	Adenocarcinoma
CC03	55	Male	Adenocarcinoma
CC04	67	Female	Adenocarcinoma
CC05	60	Male	Adenocarcinoma
CC06	74	Male	Adenocarcinoma
CC07	49	Female	Adenocarcinoma
CC08	65	Female	Adenocarcinoma
RC01	58	Male	Adenocarcinoma
RC02	63	Female	Adenocarcinoma
RC03	70	Male	Adenocarcinoma
RC04	52	Female	Adenocarcinoma
RC05	61	Male	Adenocarcinoma
RC06	47	Female	Adenocarcinoma
RC07	69	Male	Adenocarcinoma
RC08	56	Female	Adenocarcinoma

CC, Colon Cancer; RC, Rectal Cancer.

Data S1：Uncropped Western blot images corresponding to Figure S4C

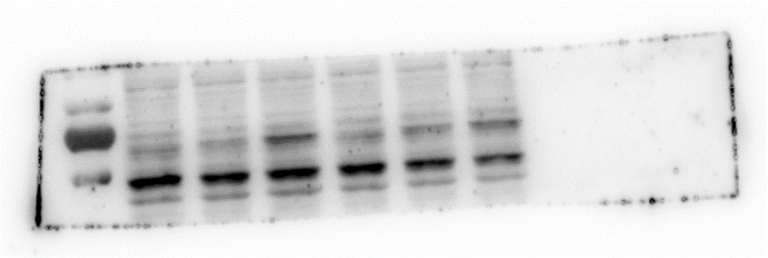

p-AKT (S473)

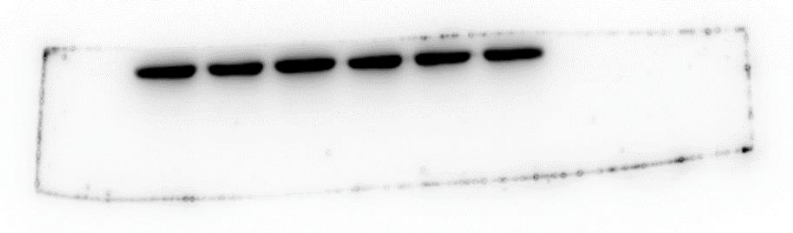

GAPDH
